# Supplementary material for: Transcriptomes of electrophysiologically recorded Dbx1-derived respiratory neurons of the preBötzinger complex in neonatal mice
Source: Sci Rep. 2022 Feb 21;12:2923. doi: 10.1038/s41598-022-06834-z (PMC8861066; doi:10.1038/s41598-022-06834-z)
Supplement: Supplementary file 1 — Supplementary Information 1. [file 41598_2022_6834_MOESM1_ESM.docx]

# Supplementary Information (Figures)


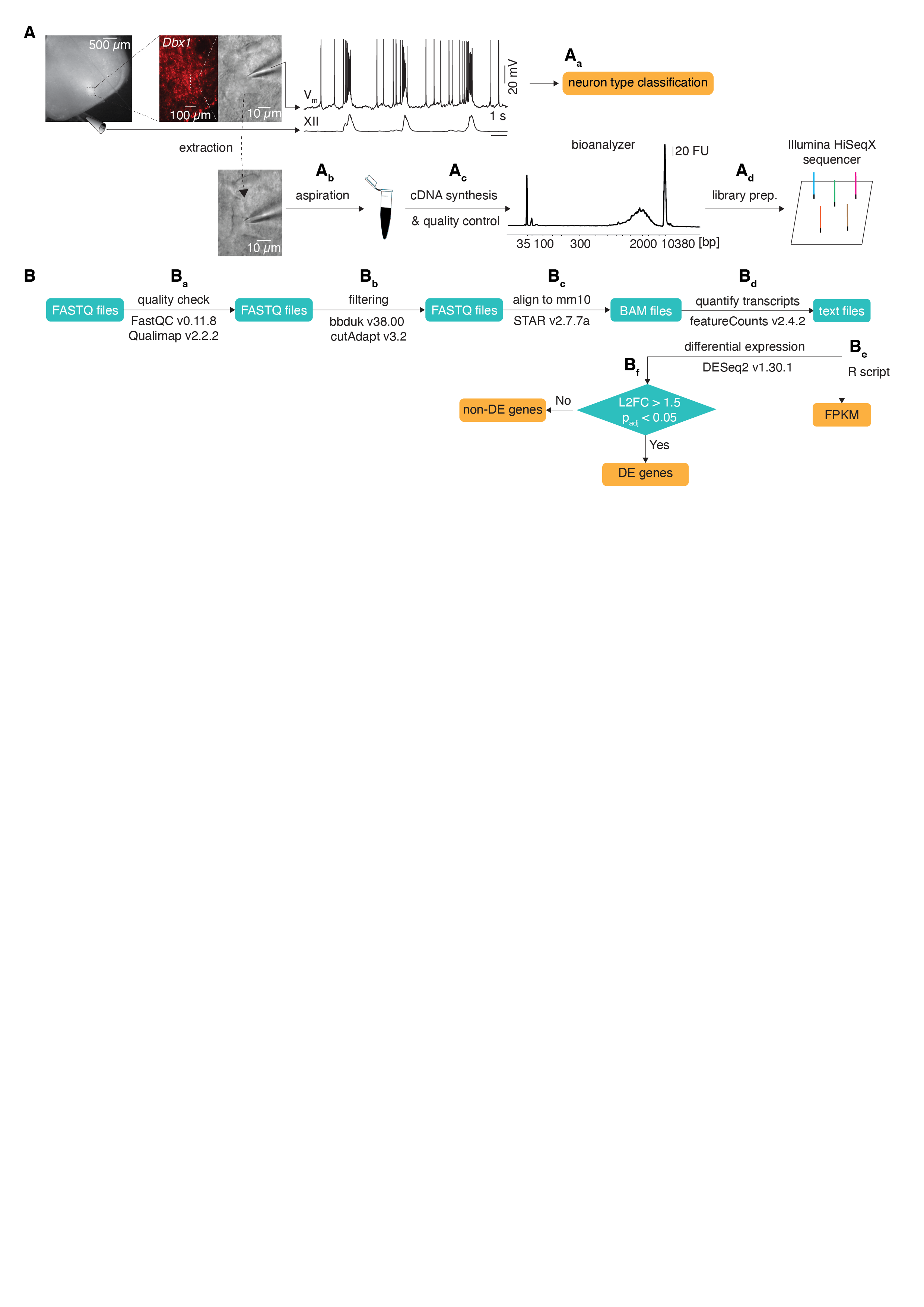


Supplementary Figure 1. Schematic explanation of Patch-Seq. **A**, Rhythmically active Dbx1 preBötC neurons identified by fluorescence and recorded in whole-cell conditions (V_m_, top trace) with XII motor output (bottom). **A_a_** – **A_d_** graphically represent steps in the Patch-Seq workflow as detailed in Material & Methods. **B**, Flowchart (**B_a_** – **B_f_**) that graphically represents the bioinformatics workflow as detailed in Materials & Methods.


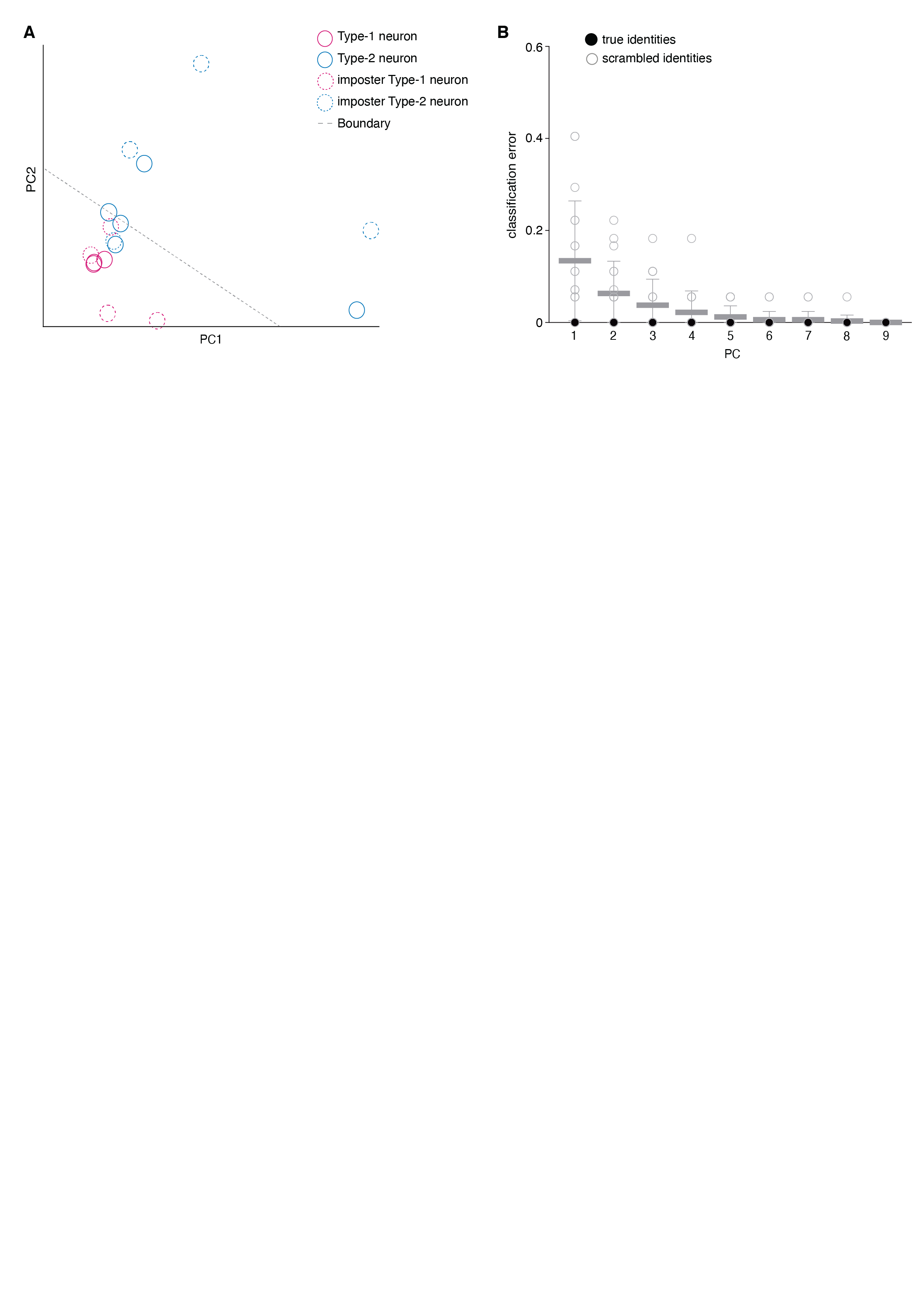


Supplementary Figure 2. Tests of PCA separation of Type-1 and Type-2 Dbx1 preBötC neurons. **A**, Example of PCA after scrambling the identities of 50% of the Dbx1 preBötC neurons as detailed in Materials & Methods. Neurons with intact identities are shown with solid circles. Neurons with scrambled identities are shown dotted-line circles. In both cases, magenta indicates Type-1 and blue-cyan indicates Type-2. **B**, Classification error (y-axis) for PCs 1-9 (x-axis) for the original data set of neurons whose identities have not been modified (filled circle) and for the surrogate data sets in which of 50% of the neurons are imposters with (unfilled gray circles). Relatively high classification errors only occur for groups that contain imposters, which bolsters confidence that Type-1 and Type-2 neurons are discrete classes.

*
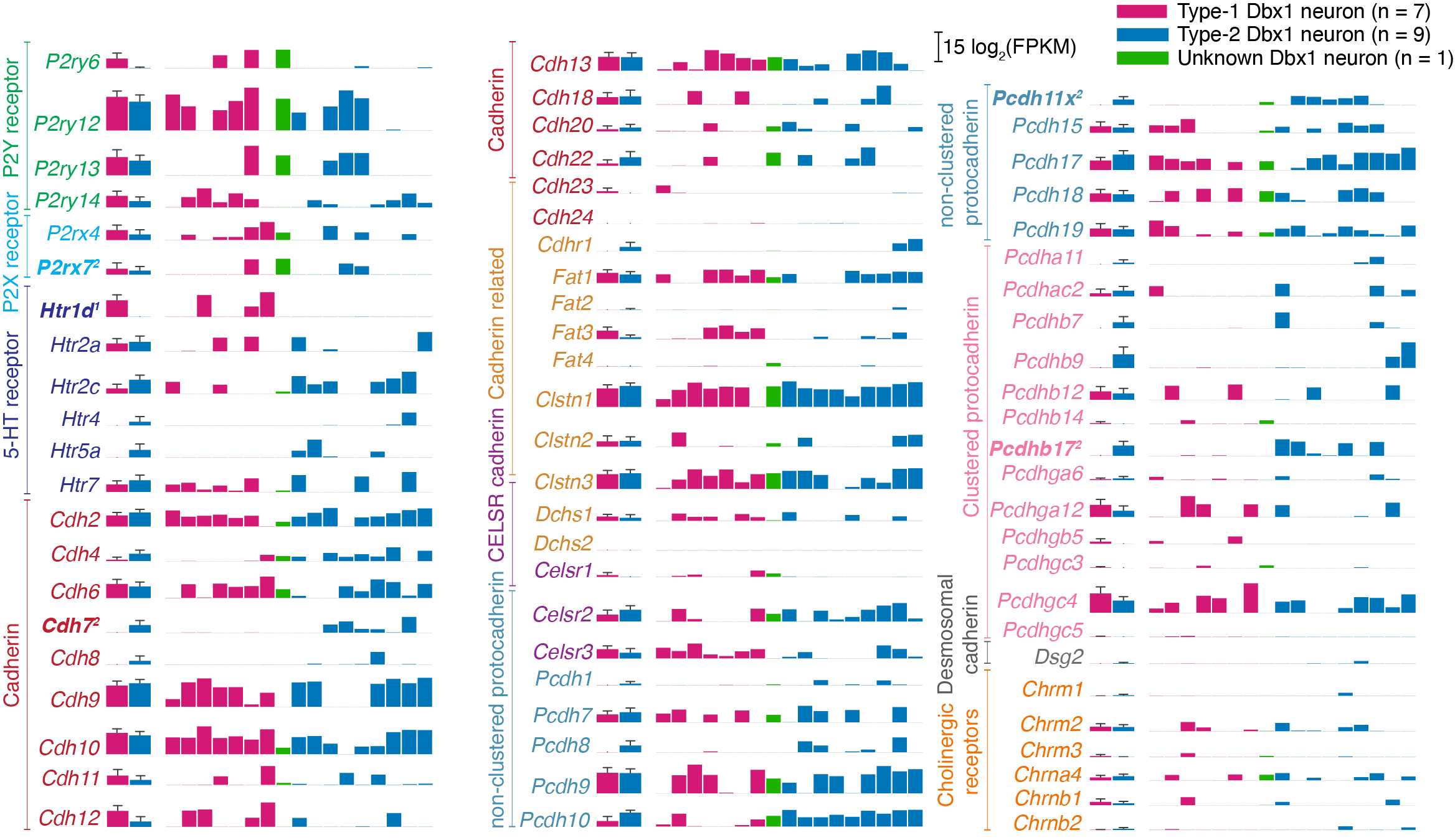
*Supplementary Figure 3. Quantitative gene expression for purinergic receptors, serotonin receptors, cadherins and cholinergic receptors. The first two bars show group data for Type-1 (n = 7; magenta bar) and Type-2 (n = 9; blue-cyan bar). The height of the bar is log_2_(mean FPKM) and the error bar with horizontal cap shows log_2_(mean + SD). The next set of 17 bars shows log_2_(FPKM) values of each neuron in the following order: 7 Type-1 neurons (magenta), 1 Unknown neuron (green), and 9 Type-2 neurons (blue-cyan). Gene names are color-coded according to the subfamily to which they belong. Gene names in bold indicate DE and contain a superscript 1 if upregulated Type-1 neurons and 2 if upregulated in Type-2 neurons.

*
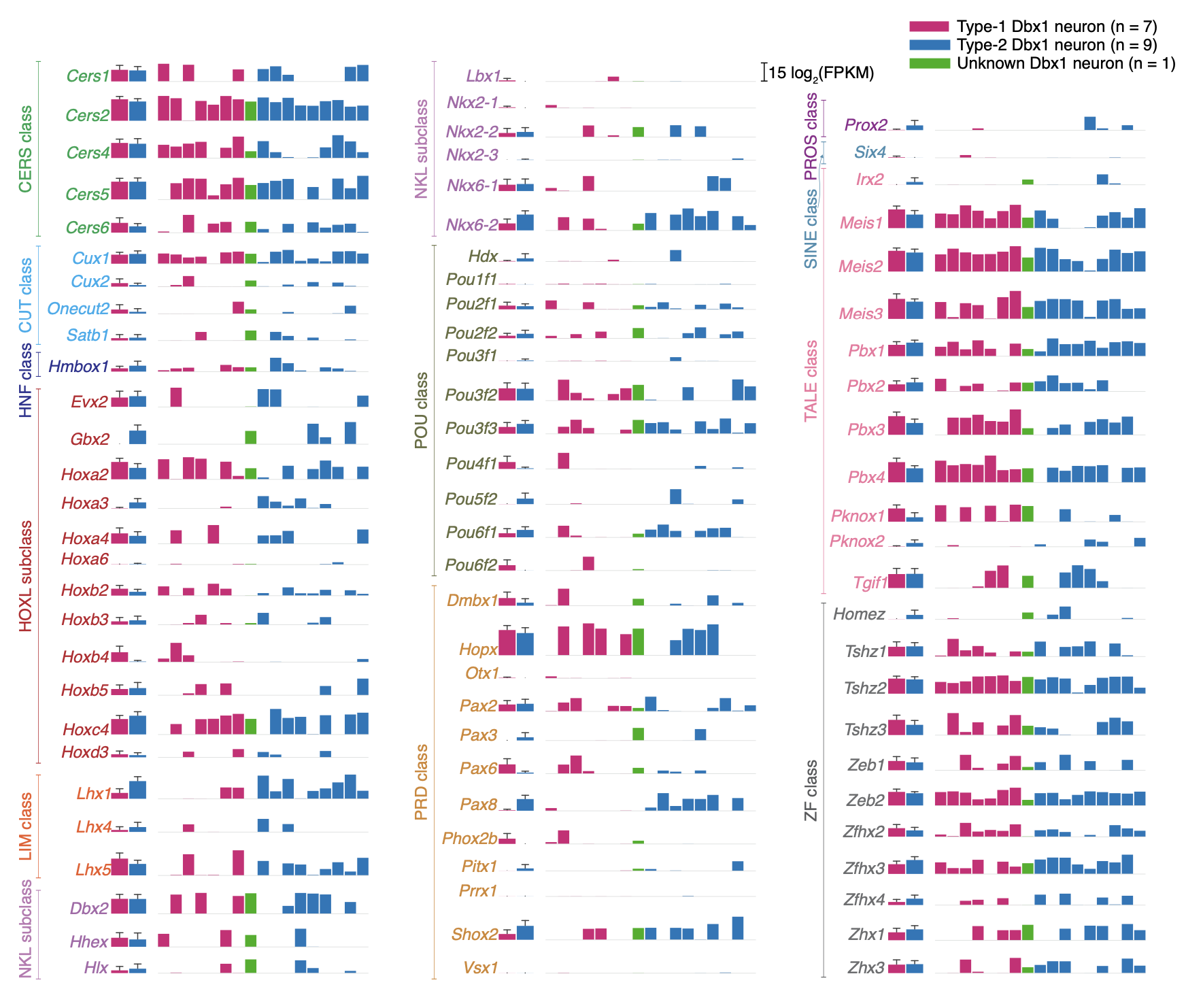
*Supplementary Figure 4. Quantitative gene expression for transcription factors. The first two bars show group data for Type-1 (n = 7; magenta bar) and Type-2 (n = 9; blue-cyan bar). The height of the bar is log_2_(mean FPKM) value and the error bar with horizontal cap shows log_2_(mean + SD). The next set of 17 bars shows log_2_(FPKM) values of each neuron in the following order: 7 Type-1 neurons (magenta), 1 Unknown neuron (green), and 9 Type-2 neurons (blue-cyan). Gene names are color-coded according to subfamily to which they belong.

# Supplementary Information (Tables)

Supplementary Table 1. **Mapping statistics of genes aligned uniquely, multimapping or unmapped to mm10 and intrinsic membrane properties of the neurons.**

Tab 1: Mapping statistics. This table lists the 15 different statistics (columns B-P) pertaining to nucleotide sequences (raw reads) mapped to the mm10 mouse genome. Each statistic is self-explanatory and listed in row 6. The bioinformatics tool used for mapping and statistical computation is listed in row 7. Sample Dbx1 preBötC neurons are listed in column A (rows 8-25). Magenta corresponds to Type-1 electrical phenotype; blue corresponds to Type-2 electrical phenotype; green corresponds to the unknown phenotype. Rows 26 and 27 provide the mean and standard deviation for the entire data set.

Tab 2: Electrical properties. This table lists the input resistance (in MΩ) and cell capacitance (in pF) for Dbx1 preBötC neurons. Sample Dbx1 preBötC neurons are listed in column A (rows 6 –22). Magenta corresponds to Type-1 electrical phenotype; blue corresponds to Type-2 electrical phenotype; green corresponds to the unknown phenotype. Supplementary Table 1 is available online as a Microsoft Excel file entitled Table_S_01_mappingStatistics.xlsx.

Supplementary Table 2. **Analyses of differential gene expression between Type-1 and Type-2 Dbx1 preBötC neurons**.

This table lists all genes evaluated by DESeq2 for differential expression between Type-1 and Type-2 Dbx1 preBötC neurons. There are 31,554 rows corresponding to every gene detected in both Types. Rows 12-134 (orange type face) list those genes that passed the stringent criteria of absolute value of the log_2_ fold-change (L2FC) exceeding 1.5 and an adjusted p-value less than 0.01. Genes are listed in column A (rows 12 – 31,554). DESeq2 statistics are listed in columns B-G. The meaning of each statistic is defined in rows 4-9. Supplementary Table 2 is available online as a Microsoft Excel file entitled Table_S_02_deseqStatistics.xlsx.

Supplementary Table 3. **Log_2_(FPKM) values of the genes that meet the inclusion criteria of gene expression in ~25% of the samples and *Oprm1*.**

This table lists all genes whose expression was detected in 25% of the samples, of any type. *Oprm1*, although not meeting this criterion, was included because of its prominent role in opioid-induced respiratory depression. Genes are listed in column A (rows 8 – 18,340). Samples are listed in columns B-V. Magenta corresponds to Type-1 electrical phenotype; blue corresponds to Type-2 electrical phenotype; green corresponds to the unknown phenotype. (Submitted as a Microsoft Excel file entitled Table_S_03_log2fpkmValues.xlsx.)
